# Demographic, health-related, and work-related factors associated with body mass index and body fat percentage among workers at six Connecticut manufacturing companies across different age groups: a cohort study

**DOI:** 10.1186/s40608-015-0073-1

**Published:** 2015-10-20

**Authors:** Jennifer L. Garza, Alicia G. Dugan, Pouran D. Faghri, Amy A. Gorin, Tania B. Huedo-Medina, Anne M. Kenny, Martin G. Cherniack, Jennifer M. Cavallari

**Affiliations:** Division of Occupational and Environmental Medicine, UConn Health, 263 Farmington Ave, Farmington, CT 06030 USA; Department of Allied Health Sciences, University of Connecticut, 358 Mansfield Road, Unit 1101, Storrs, CT 06269 USA; Department of Psychology, University of Connecticut, 2006 Hillside Road, Unit 1248, Storrs, CT 06269 USA; Department of Statistics, UConn Health, 263 Mansfield Road, Unit 1101, Storrs, CT 06269 USA; Department of Community Medicine and Health Care, UConn Health, 263 Mansfield Road, Unit 1101, Storrs, CT 06269 USA; Geriatric Medicine, UConn Health, 263 Farmington Ave, Farmington, CT 06030 USA; Department of Community Medicine, UConn Health, 263 Farmington Ave, Farmington, CT 06030 USA

**Keywords:** Occupational, Obesity, Workplace, Age

## Abstract

**Background:**

Effective workplace interventions that consider the multifactorial nature of obesity are needed to reduce and prevent obesity among adults. Furthermore, the factors associated with obesity may differ for workers across age groups. Therefore, the objective of this study was to identify demographic, health-related, and work-related factors associated with baseline and changes in body mass index (BMI) and body fat percentage (BFP) and among Connecticut manufacturing workers acrossage groups.

**Methods:**

BMI and BFPof 758 workers from six Connecticut manufacturing companies were objectively measuredat two time points approximately 36 months apart. Demographic, health-related, and work-related factors wereassessed via questionnaire. All variables were included in linear regression models to identify factors associated with baseline and changes in BMI and BFP for workers in 3 age groups: <45 years (35 %), 45–55 years (37 %), >55 years (28 %).

**Results:**

There were differences in baseline and changes in BMI and BFP among manufacturing workers across age groups. Being interested in changing weight was significantly (*p* < 0.01) associated with higher baseline BMI and BFP across all age categories. Other factors associated with higher baseline BMI and BFP differed by age group and included: male gender (BMI *p* = 0.04), female gender (BFP p < 0.01), not having a college education (BMI *p* = 0.01, BFP *p* = 0.04), having childcare responsibilities (BMI *p* = 0.04), and working less overtime (*p* = 0.02) among workers in the <45 year age category, male gender (BMI *p* = 0.02), female gender (BFP *p* < 0.01) and reporting higher stress in general (BMI *p* = 0.04) among workers in the 45–55 year age category, and female gender (BFP *p* < 0.01) and job tenure (BFP *p* = 0.03) among workers in the >55 year age category. Few factors were associated with change in BMI or BFP across any age category.

**Conclusions:**

Among manufacturing workers, we identified associations between individual, health-related, and work-related factors and baseline BMIand BFP that differed by age. Such results support the use of strategies tailored to the challenges faced by workers in specific age groups rather than adopting a one size fits all approach. Effective interventions should consider a full range of individual, health-related, and work-related factors. More work must be done to identify factors or strategies associated with changes in obesity over time.

## Background

Obesity can have serious adverse health consequences including early death andheart disease [1]. Therefore, with almost 70 % of American adults at an unhealthy body mass index [2], interventions to support healthy eating, exercise, and weight loss have become increasingly commonplace. Previous interventions addressing obesity have primarily focused on encouraging health-related behavioral changes such as to diet or physical activity among participants, without taking into account other factors that may be contributing to the problem [3–7]. Yet, a variety of other factors are known to affect obesity. The Social Ecological Model, which emphasizes the relationships among multiple factors affecting health, can be applied to the study of obesity [8]. Studies have reported associations between demographic factors such as education, relationship status, and socioeconomic status and obesity [9–11]. Work-related factors such as job stress, long working hours, and shift work have also been associated with obesity [12–14]. Demographic or work-related factors can affect obesity through many pathways from directly influencing physiology to influencing diet or physical activity [15]. For example, chronic exposure to stress at work can result in neuroendocrine dysregulation [16], and may also lead to unhealthy behaviors [17].

Effective obesity interventions should consider the demographic, health-related, and work-related factors that are most relevant to the target population. The factors most strongly associated with obesity may differ for groups of individualsacross industries or age categories. For example, Parkes [18] identified associations between education and marital status and body mass index (BMI) among offshore oil industry workers, while Duffy et al. [19] did not find associations between either of these factors and BMI among operating engineers but instead identified other factors that were associated with BMI in this population. Across industries, workershave exposures to different factors such as psychological or physical job demands that may contribute to weight gain [20]. Both Parkes and Duffy et al. also identified age as being associated with BMI, with Parkes observing an interaction between age and physical work demands where older offshore oil industry workers with more physically demanding jobs had greater declines in BMI than other workers [18, 19]. Individuals across ages may have different home or work demands and responsibilities that could affect obesity [21, 22]. Recognizing the industry and age-specific factors that contribute to obesity will allow for intervention strategies that are more relevant and perhaps more successful.

The objective of this study was to identify demographic, health-related, and work-related factors associated with BMI and body fat percentage (BFP) among manufacturing workers of different ages. The results of this study maybe used to inform interventions around obesity.

## Methods

### Study design and participants

This study is part of a large longitudinal cohort study of six medium-sized manufacturing companies in Connecticut, designed to assess changes over time in an aging workforce, focusing in particular on musculoskeletal, psychosocial, and work-related variables. The full study protocol was approved by University of Connecticut Health Center’s Institutional Review Board. Eligibility criteria for study sites were: medium company size; broad age distribution centered on late 5th and 6th decades, and a workforce engaged in skilled light-manufacturing with high degrees of repetition. Four of the organizations had labor unions. Details of site identification and study procedures at each company are available in a prior publication [23].

The current study used data on BMI and BFP collected from physical performance testing performed at two time points, time 1 and time 2, approximately 36 months apart (average time between collections 33 months), and demographic, health-related, and work-related factors collected from paper-and-pencil surveys conducted at time 1. During the workday, following informed consent, surveys were distributed and collected by members of the research team. Participants were given a small financial incentive for completing the survey or physical testing measurements. All employees at selected sites were considered eligible and invited to participate in the study; no exclusion criteria were specified. Employees of all job classifications participated (e.g., production, sales, administrative, managerial staff).

### BMI and BFP

BMI was calculated based on objective measurements of each participant’s height and weight. A vertical anthropometer was used to measure height in centimeters. Participants were barefoot for measurement. Weight was determined with the use of a standard balance scale with the balance was calibrated to zero. Values for height were recorded to the nearest tenth of a centimeter, and weight was recorded to the nearest quarter kilogram [24].

BFP was estimated through bioelectrical impedance [25, 26]. A Bioelectrical Body Composition Analyzer (Quantum X, RJL Systems, Clinton Township, MI) captured reactance and resistance for conversion to proportional body fat content. All testing was performed in accordance with the manufacturer’s instruction: shoes, socks, and jewelry or clothes with metal appurtenances were removed, and subjects were supine for 5 min prior to testing.

### Demographic, health-related, and work-related factors

Demographic variables included age, gender, race (White/European Descent, Black/African American/African, American Indian/Alaska Native, Asian /Asian American. Other), marital status (married or live with partner, widowed, divorced or separated, single or never married), education level (less than high school, high school graduate or GED, some college, 2 or 4 year college degree, graduate degree),family income ($10,000–24,999, $25,000–49,999, $50,000–74,999, $75,000–99,999, More than $100,000), childcare responsibility, and elder care responsibility. Childcare responsibility was measured with one question: “How much responsibility do you personally have for any children under 18 in your household?” Respondents checking that they had primary or shared responsibility were defined as having a high level of childcare responsibility, while those who indicated that they had no children under 18 at home or that another adult had primary responsibility were defined as having a low level of childcare responsibility. Elder care responsibility was measured with one question: “How many adults age 65 and older depend on you in any way to help them due to disability or chronic illness? “Respondents checking 1 or greater were defined as providing elder care, while those responding “zero” were defined as not providing elder care.

We examined eight health-related factors including hours of sleep, depressive symptoms, leisure time physical activity, musculoskeletal pain, weight perception, and work-life balance. Hours of sleep was assessed with a single-item measure from the Pittsburgh Sleep Quality Index that asked: “During the work week, about how many hours of sleep do you typically get per 24-h period?”[27]. There were eight response options (<4 h, 4–5 h, 5–6 h, 6–7 h, 7–8 h, 8–9 h, 9–10 h, > 10 h). Depressive symptoms were assessed with an 8-item version of the CES-D scale, which has shows excellent reliability in studies of adults ([28]; α = .80). The measure listed several symptoms of depression (e.g., sad, lonely) and asked respondents how often they experienced each symptom on a 4-point rating scale from 0 (less than 1 day per week) to 3 (5–7 days per week); scores are calculated by summing across the item ratings. Leisure time physical activity was assessed with one item: “Outside of work, in an average week during the past year, how many hours did you spend on… physical exercise such as fitness, aerobics, swimming, jogging, cycling, tennis, etc.?” adapted from the EPIC Physical Activity Questionnaire [29]. Response options included: 0 h per week, 1–3 h per week, 4–6 h per week, 7–9 h per week, 10–12 h per week, greater than 12 h per week.

Musculoskeletal pain was assessed with the question: “During the past 3 months, how much pain, aching or stiffness/limited motion have you had in the areas shown on the diagram below?”[30, 31]. The measure listed seven areas of the musculoskeleture (e.g., low back, knee) and asked respondents to rate how severely each area was affected on a 5-point rating scale from 0 (mild) to 4 (extreme). Participants were considered to have musculoskeletal pain if they indicated a score of 2 (moderate) or more in any body area. Weight perception was assessed with one item: “Tell us whether you are interested in making changes or improvements in your health in the following area… lose weight or maintain healthy weight”[30]. Response options were: 0 (not interested in changing), 2 (interested in changing), and 3 (currently doing this to my satisfaction). Work-life balance was based on one question, “How successful do you feel at balancing your paid work and your family life? Do you feel…?” Response options ranged on a 5-point scale from 1 (not at all successful) to 4 (completely successful) [32].

We examined ten work-related factors including job tenure, job type, work shift, overtime, time standing at work, job satisfaction, civility norms, decision latitude, procedural justice, psychological demands, social support, and stress in general. Job tenure was assessed with the open-ended question “How many years have you worked at your organization?” to which respondents entered a numeral. Job type was measured with an item to assess whether employees were either production workers on the shop floor or administrative employees in office jobs (i.e., managers, sales and administrative staff); each job type places distinct biomechanical and psychosocial demands on workers. Work shift was measured using one question “What shift do you typically work?” with three possible response options (firstshift, second shift, third shift). Work overtime was assessed with one question “Thinking of the past year, which best describes the amount of overtime or extra hours you work in an average month?” that had six response options (0–4 h, 5–12 h, 13–24 h, 25–36 h, 37–50 h,51 h and above). Work time standing was measured with one question: “Please check the box that best describes how much standing/walking you do on your job, from always sitting (0 %) to always standing or walking (100 %)” followed by 11 response options (0 % always sitting, 10, 20, 30, 40, 50 % Half & Half, 60, 70, 80 90, 100 % always standing or walking).

Job satisfaction was assessed using a 3-item measure [33]; a sample item was “I am satisfied with the overall quality of work done in my workgroup” to which participated responded using a 5-point scale that ranged from 1 (strongly disagree) to 5 (strongly agree) and a score was calculated by averaging ratings across the items. Civility norms was assessed using a 4-item measure [34]; a sample item was “Respectful treatment is the norm in my department” to which participated responded using a 5-point scale that ranged from 1 (strongly disagree) to 5 (strongly agree) and a score was calculated by averaging ratings across the items. Decision latitude was measured with a subscale from the job content questionnaire [35] consisting of seven items that assess skill discretion and decision authority. Sample items include: “My job requires me to be creative,” and “My job allows me to make a lot of decisions on my own.” Response options ranged on a 4-point scale from 1 (strongly disagree) to 4 (strongly agree) and a score was calculated by averaging ratings across the items. Procedural justice was measured with four items [36] that assess work experiences. A sample item is: “Job decisions are made in an unbiased manner.” Response options ranged on a 5-point scale from 1 (strongly disagree) to 5 (strongly agree) and a score was calculated by averaging ratings across the items. Psychological job demands were assessed with a subscale from the job content questionnaire [35]. A sample item was: “My job requires working very hard.” Response options ranged on a 4-point scale from 1 (strongly disagree) to 4 (strongly agree) and a score was calculated by averaging ratings across the items. Stress was assessed with a six-item version of the Stress in General scale (SIG; [37]; α = .91), which instructs respondents to indicate whether several words or phrases describe their work (e.g., irritating, hectic, hassled). Each item was rated with a 0 (no), 1.5 (cannot decide), or 3 (yes), and a score was calculated by averaging ratings across the items. Social support was measured with a subscale from the Job Content Questionnaire (JCQ: [35]) consisting of four items that assess instrumental and socioemotional social support from supervisors and coworkers including “(My supervisor is)/(People I work with are) helpful in getting the job done” and “(My supervisor/People I work with) take a personal interest in me”. Response options ranged on a 4-point scale from 1 (strongly disagree) to 4 (strongly agree) and a score was calculated by averaging ratings across the items.

### Data analysis

BMI and BFP were treated as continuous variables for all analyses. Age was grouped into three categories (under 45 years old, 45–54 years old, 55 or more years old) with about a third of the sample in each group. All other demographic, health-related, and work-related factors were dichotomized in order to reduce the number of degrees of freedom to be included in the models. When dichotomizing variables, we aimed to choose standard cutoffs or to divide data into two categories as equally distributed as possible in order to optimize power.

Demographic variables dichotomized included race (white, other), marital status (married or living with partner, other), education level (at least some college, no college), family income (less than $75,000, $75,000 and over), childcare responsibility (some or complete responsibility, none or another adult responsible), and eldercare responsibility (responsible for at least one adult, no responsibility).

Health-related variables that were dichotomized included sleep hours (less than 6 h, 6 or more hours), depressive symptoms (1 day per week or less, more than 1 day per week), leisure time physical activity (at least some, none), musculoskeletal pain (none to mild, moderate to severe), stress (low, high), weight perception (interested in changed, not interested), work-life balance (not or somewhat successful, very or completely successful), and social support (disagree, agree).

Work-related variables that were dichotomized included job tenure (five years or more, less than 5 years), work shift (first shift, other), overtime (less than 24 h per month, 24 h per month or more), time standing at work (standing 30 % of the time or less, standing more than 30 % of the time), job satisfaction (agree, neutral/disagree), civility norms (agree, neutral/disagree), decision latitude (agree,disagree), procedural justice (agree, neutral/disagree), and psychological demands (agree,disagree).

We used chi-squared tests to evaluate differences in the distribution of factors, BMI, and BFP by age. To identify factors associated with BMI and BFP, we performed multivariate linear regression analyses, stratified by age, using all demographic, health-related, and work-related factors to assess associations with baseline and change in BMI and BFP. Before performing the multivariate analyses, we used kappa tests to assess correlation among demographic, health-related, and work-related factors, but because no factors were highly correlated (kappa coefficient > 0.7), we did not restrict the factors included in the multivariate regression models. All statistical analyses were performed in SAS version 9.4 (Cary, NC). Significance was defined as two-tailed *p* < 0.05.

## Results

A total of 758 participants ranging in age from 20–71 years old were included in this study. The population was categorized into similarly sized age categories with 35 % aged <45 years, 37 % aged 45–55 years, and 28 % aged > 55 years (Table 1). The manufacturing workplace consisted predominantly of white males (Table 1). More workers 45 and over were married while fewer were college educated. Childcare and eldercare responsibilities differed by age, with the largest percentage of workers having childcare responsibilities aged <45 years (57 %) and the largest percentage of workers having elder care responsibilities aged >55 years (36 %) (Table 1). The only health-related factors that had different distributions across age categories were the amount of leisure time physical activity, which was lowest among workers <45 years and work-life balance which was most successful among workers >55 years old (Table 1). As would be expected, job tenure and the percentage of administrative jobs increased with age and the percentage of time standing decreased with age (Table 1). A higher percentage of workers >55 years reported high job satisfaction (Table 1).

**Table 1:** Distribution of demographic, health-related, and work-related factors by age

	<45 years		4555 years		>55 years	
	N	Percent	N	Percent	N	Percent
Population (*n* = 758)	269	35	277	37	212	28
Demographics						
Gender						
Male	203	75	191	69	142	67
Female	66	25	86	31	70	33
*p*-value	0.09					
Race						
White	210	78	242	87	177	83
Other	59	22	36	13	35	17
*p*-value	0.02					
Marital Status						
Married or living with partner	155	58	218	79	166	79
Other	114	42	58	21	45	21
*p*-value	<0.01					
Education						
At least some college	196	74	157	57	117	56
No college	69	26	118	43	93	44
*p*-value	<0.01					
Family Income						
> = $75,000/year	154	57	181	66	127	63
<$75,000/year	115	43	92	34	76	37
*p*-value	0.09					
Childcare Responsibilities						
Some or Complete Responsibility	154	57	130	47	24	11
None or Another Adult Responsible	115	43	144	53	186	89
*p*-value	<0.01					
Elder Care Responsibility						
Responsible for at least one adult	55	20	102	37	77	36
None	214	80	176	63	135	64
*p*-value	<0.01					
Health-Related Factors						
Hours Sleep						
> = 6 h/night	163	61	168	61	140	67
<6 h/night	105	39	109	39	70	33
*p*-value	0.32					
Depressive Symptoms						
<=1 day/week	22	9	28	11	22	12
>1 day/week	233	91	234	89	166	88
*p*-value	0.54					
Leisure Time Physical Activity						
At least some	203	76	200	72	131	62
None	64	24	77	28	79	38
*p*-value	<0.01					
Musculoskeletal Pain						
None or Mild	152	57	146	53	108	51
Moderate to Severe	117	43	132	47	103	49
*p*-value	0.46					
Weight Perception						
Interested in Changing	153	57	181	65	129	61
Other	114	43	96	35	81	39
*p*-value	0.16					
Work-Life Balance						
Very/Completely Successful	93	35	105	38	98	46
Not/Somewhat Succesful	174	65	173	62	113	54
*p*-value	0.03					
Work-Related Factors						
Job Tenure						
> = 5 years	130	49	233	84	185	89
<5 years	138	51	45	16	23	11
*p*-value	<0.01					
Job Type						
Administrative	95	37	113	42	106	55
Floor	159	63	154	58	85	45
*p*-value	<0.01					
Work Shift						
First	189	70	213	77	161	76
Other	80	30	65	23	51	24
*p*-value	0.19					
Work Overtime						
<24 h/month	177	66	182	66	142	68
> = 24 h/month	91	34	95	34	68	32
*p*-value	0.9					
Work Time Standing						
>30 % of time	167	63	192	69	154	74
<=30 % of time	99	37	85	31	53	26
*p*-value	0.02					
High Job Satisfaction						
Agree (>3)	154	57	176	64	149	70
Neutral/Disagree (<=3)	115	43	101	36	63	30
*p*-value	0.01					
High Civility Norms						
Agree (>3)	183	68	190	69	164	77
Neutral/Disagree (<=3)	86	32	86	31	48	23
*p*-value	0.05					
High Decision Latitude						
Agree (> = 3)	127	47	139	50	109	52
Disagree (<3)	142	53	139	50	102	48
*p*-value	0.61					
High Procedural Justice						
Agree (>3)	124	46	103	37	98	47
Neutral/Disagree (<=3)	145	54	174	63	111	53
*p*-value	0.05					
High Psychological Demands						
Agree (> = 3)	80	30	75	27	45	21
Disagree (<3)	189	70	203	73	165	79
*p*-value	0.12					
High Social Support						
Agree (> = 3)	156	58	148	53	132	63
Disagree (<3)	113	42	130	47	79	37
*p*-value	0.12					
Stress in General						
Low (<=1.5)	169	63	167	60	144	68
High (>1.5)	100	37	110	40	79	37
*p*-value	0.19					

The distribution of BMI and BFP and the change in BMI and BFP over a 33 month period is presented for each of the three age categories (Table 2). There were significant differences in baseline (*p* = 0.04) and change in BMI (*p* < 0.01) by age, with the >55 year age group having larger mean baseline BMI’s (29.7 compared to 28.7 for the <45 year age group) but also experiencing negative changes (decreases) in BMI from baseline to time 2 (−0.4 compared to 0.1 for the <45 year age group and 0.3 for the 45–55 year age group). There was also a significant (*p* < 0.01) difference in baseline BFP by age, with participants in the <45 year age group having the lowest baseline BFPs (26.0 compared to 28.1 for the 45–55 year age group and 28.6 for the >55 year age group). We did not observe significant differences in change in BFP by age (*p* = 0.08).

**Table 2:** Differences in body mass index (BMI) and body fat percentage (BFP) by age

	<45 years	45–55 years	>55 years	
	Mean (SD)	Mean (SD)	Mean (SD)	*p*-value
Body Mass Index				
Time 1	28.7 (5.9)	29.8 (5.3)	29.7 (4.7)	0.04
Change	0.1 (2.0)	0.3 (1.8)	-0.4 (2.0)	<0.01
Body Fat Percent (%)				
Time 1	26.0 (8.4)	28.1 (7.9)	28.6 (8.3)	<0.01
Change	-0.4 (5.2)	0.2 (4.2)	-1.0 (4.1)	0.08

### <45 year age category factors, baseline BMI and BFP

**Fig. 1:**
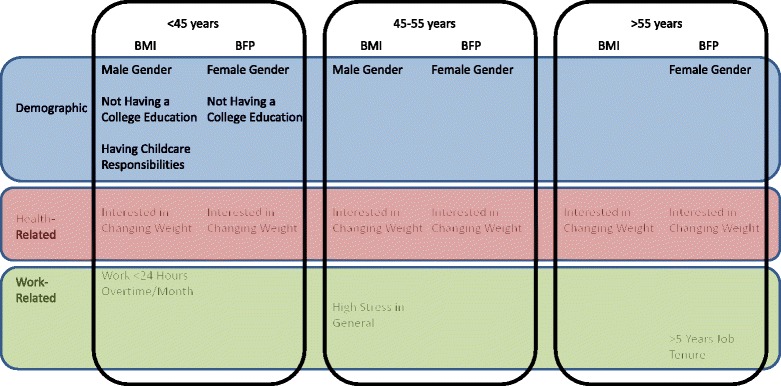
Factors associated with baseline BMI and BFP across age groups

Factors associated with baseline BMI and BFP levels are presented for each age category in Table 3 and summarized in Fig. 1. In the <45 year age group, demographic factors associated with baseline BMI and BFP were similar including gender and education. Workers <45 years with childcare responsibilities had a significantly (*p* = 0.04) higher baseline BMI as compared to workers with no responsibilities and BFP was also higher, although not statistically significant (*p* = 0.42). The only health-related factor that was significantly associated with both baseline BMI and BFP in the <45 year age category was being interested in changing weight (*p* < 0.01). In the <45 year age category, work-related factors associated with BMI included working overtime, where workers who worked >24 h/month of overtime had lower BMI (*p* = 0.02) and a trend towards lower BFP, although not significantly (*p* = 0.12).

**Table 3:** Multivariate analyses of the relationship between demographic, health related, and work related factors and time 1 BMI and BFP

	<45 years						45–55 years						>55 years					
	BMI			BFP			BMI			BFP			BMI			BFP		
	β	(SE)	*p*-value	β	(SE)	*p*-value	β	(SE)	*p*-value	β	(SE)	*p*-value	β	(SE)	*p*-value	β	(SE)	*p*-value
Demographics																		
Gender																		
Male (Ref)																		
Female	−1.91	(0.9)	0.04	9.0	(1.2)	<0.01	−1.88	(0.8)	0.02	10.27	(1.0)	<0.01	0.01	(1.0)	0.99	10.75	(1.3)	<0.01
Race																		
White (Ref)																		
Other	0.17	(0.8)	0.84	0.17	(1.1)	0.87	0.65	(1.1)	0.56	2.52	(1.3)	0.06	1.22	(1.2)	0.3	1.88	(1.5)	0.22
Marital Status																		
Married or living with partner (Ref)																		
Other	0.49	(0.9)	0.57	1.28	(1.1)	0.26	0.55	(0.9)	0.54	0.32	(1.0)	0.75	0.11	(1.1)	0.92	−0.62	(1.5)	0.67
Education																		
At least some college (Ref)																		
No college	2.44	(1.0)	0.01	2.52	(1.2)	0.04	0.48	(0.8)	0.55	1.41	(0.9)	0.13	0.53	(1.0)	0.59	−1.27	(1.3)	0.34
Family Income																		
> = $75,000/year (Ref)																		
<$75,000/year	0.66	(0.9)	0.44	−0.09	(1.1)	0.93	0.87	(0.8)	0.3	1.15	(1.0)	0.24	−0.22	(1.1)	0.84	1.16	(1.4)	0.41
Childcare Responsibilities																		
Some or Complete Responsibility (Ref)	1.7	(0.8)	0.04	0.84	(1.0)	0.42	−0.92	(0.7)	0.18	−0.39	(0.8)	0.62	−1.96	(1.2)	0.11	−1.59	(1.6)	0.33
None or Another Adult Responsible																		
Adult Care Responsibility																		
Responsible for at least one adult (Ref)	−0.34	(0.9)	0.71	−1.44	(1.1)	0.2	0.67	(0.7)	0.35	0.31	(0.8)	0.7	0.36	(0.8)	0.67	−0.34	(1.1)	0.75
None																		
Health-Related Factors																		
Depressive Symptoms																		
<=1 day/week (Ref)																		
>1 day/week	−1.99	(1.3)	0.14	0.49	(1.7)	0.77	0.31	(1.2)	0.79	0.66	(1.3)	0.62	−0.29	(1.4)	0.83	0.58	(1.8)	0.75
Hours Sleep																		
> = 6 h/night (Ref)																		
<6 h/night	−0.1	(0.8)	0.9	0.13	(1.0)	0.89	0.9	(0.8)	0.23	0.81	(0.9)	0.35	0.06	(0.9)	0.94	0.25	(1.1)	0.82
Leisure Time Physical Activity																		
None (Ref)																		
At least some	−0.26	(0.9)	0.76	0.31	(1.1)	0.77	0.75	(0.8)	0.36	1.7	(0.9)	0.07	0.87	(0.8)	0.28	0.97	(1.1)	0.37
Musculoskeletal Pain																		
None or Mild (Ref)																		
Moderate to Severe	1.01	(0.7)	0.17	0.75	(0.9)	0.42	1.2	(0.7)	0.1	0.84	(0.8)	0.31	0.53	(0.8)	0.52	0.3	(1.1)	0.78
Weight Perception																		
Interested in Changing (Ref)																		
Other	−5.04	(0.7)	<0.01	−7.07	(0.9)	<0.01	−2.96	(0.8)	<0.01	−3.24	(0.9)	<0.01	−5.51	(0.9)	<0.01	−5.26	(1.1)	<0.01
Work-Related Factors																		
Civility Norms																		
High (>4) (Ref)																		
Low (<4)	1.3	(0.9)	0.14	−1.76	(1.1)	0.1	0.28	(0.8)	0.73	−0.27	(0.9)	0.77	0.74	(1.0)	0.47	−0.42	(1.3)	0.75
Decision Latitude																		
Low (<3) (Ref)																		
High (>3)	0.11	(0.9)	0.9	−1.2	(1.0)	0.25	−0.27	(0.9)	0.75	−0.87	(0.9)	0.35	0.71	(1.0)	0.46	0.38	(1.3)	0.76
Job Satisfaction																		
High (>4) (Ref)																		
Low (<4)	1.37	(0.8)	0.11	−1.0	(1.0)	0.31	−0.09	(0.8)	0.91	0.45	(0.9)	0.63	−1.41	(1.0)	0.15	−1.18	(1.3)	0.36
Job Tenure																		
>5 years (Ref)																		
<5 years	−0.83	(0.8)	0.27	−0.66	(1.0)	0.49	−0.29	(0.9)	0.76	−0.72	(1.1)	0.51	−1.15	(1.2)	0.35	−3.59	(1.6)	0.03
Job Type																		
Administrative (Ref)																		
Floor	−0.39	(0.9)	0.66	−1.36	(1.1)	0.21	−0.17	(0.8)	0.82	−0.1	(0.9)	0.9	−0.02	(0.9)	0.98	−1.57	(1.1)	0.16
Procedural Justice																		
High (>4)																		
Low (<4)	1.29	(1.4)	0.34	1.53	(1.0)	0.14	−0.42	(1.5)	0.78	0.27	(0.9)	0.77	−1.3	(1.5)	0.38	1.1	(1.2)	0.36
Psychological Demands																		
Low (<3) (Ref)																		
High (>3)	0.78	(1.2)	0.51	−1.52	(1.1)	0.17	0.68	(1.0)	0.52	−0.08	(0.9)	0.94	0.18	(1.6)	0.91	−1.37	(1.4)	0.33
Social Support																		
Low (<3) (Ref)																		
High (>3)	1.98	(1.0)	0.05	1.83	(1.0)	0.08	0.09	(1.0)	0.93	0.52	(0.9)	0.56	−1.05	(1.1)	0.33	−1.19	(1.1)	0.29
Stress in General																		
Low (<1.5) (Ref)																		
High (>1.5)	1.18	(0.8)	0.15	0.6	(1.1)	0.59	1.52	(0.7)	0.04	1.28	(0.9)	0.15	0.67	(0.9)	0.45	−0.24	(1.3)	0.85
Work-Life Balance																		
Very/Completely Successful (Ref)																		
Not/Somewhat Successful	−0.45	(0.8)	0.59	−0.63	(1.1)	0.55	−0.79	(0.8)	0.33	−0.03	(0.9)	0.98	−0.16	(0.9)	0.86	1.52	(1.2)	0.19
Work Overtime																		
<24 h/month (Ref)																		
>24 h/month	−2.02	(0.9)	0.02	−1.65	(1.1)	0.12	−0.14	(0.7)	0.85	0.55	(0.9)	0.52	0.92	(0.9)	0.32	0.42	(1.2)	0.73
Work Shift																		
First (Ref)																		
Other	1.25	(1.0)	0.21	0.99	(1.2)	0.42	0.18	(0.9)	0.84	−0.36	(1.0)	0.72	−0.76	(1.1)	0.47	−0.62	(1.4)	0.66
Work Time Standing																		
>30 % of time (Ref)																		
<30 % of time	−1.08	(0.8)	0.18	−1.7	(1.0)	0.1	−0.18	(0.8)	0.82	1.48	(1.0)	0.12	−1.18	(0.9)	0.19	−0.83	(1.2)	0.47

### 45–55 year age category factors, baseline BMI and BFP

In the 45–55 year age category, male gender was significantly associated with increased baseline BMI, yet decreased BFP (Table 3). The only health-related factor associated with baseline BMI and BFP in 45–55 year age category was interest in changing weight, which was associated with a statistically significantly (*p* < 0.01) increased baseline BMI and BFP (Table 3). The only work-related factor associated with significantly increased baseline BMI in the 45–55 year age category washigh stress, which was also associated with a trend towards higher baseline BFP, although the relationship was not statistically significant (*p* = 0.15).

### >55 year age category factors, baseline BMI and BFP

In the >55 year age category, while there was no association between gender and baseline BMI, women had significantly (*p* < 0.01) higher baseline BFP as compared to men (Table 3). Being interested in changing weight was the only health-related factor significantly (*p* < 0.01) associated with higher baseline BMI and BFP among participants in the >55 year age group (Table 3). For work-related factors, only longer job tenure was significantly (*p* = 0.03) associated with increased baseline BFP.

### Change in BMI and BFP

The factors associated with changes in BMI and BFP over a 33 month time period are presented by age category in Table 4. Few factors were associated with changes. In the <45 year age category, those with no college education experienced a significant (*p* = 0.04) decreased BMI (Table 4). Within this same age category, elder care responsibilities were associated with a significantly (*p* = 0.02) increased BFP (Table 4). In the 45–55 year age group, no factors were significantly associated with change in BMI among participants. Yet, in this 45–55 year age group, some work-related factors were associated with changes in BFP. Significant increases in BFP were observed among workers with low job satisfaction (*p* = 0.02) and working >24 h per month overtime (*p* = 0.04); high job demands was significantly (*p* = 0.04) associated with decreased BFP. There were no significant factors associated with change in BMI or BFP among participants in the >55 age group.

**Table 4:** Multivariate analyses of the relationship between demographic, health related, and work related factors and change in BMI and BFP

	<45 years						45–55 years						>55 years					
	BMI			BFP			BMI			BFP			BMI			BFP		
	β	(SE)	*p*-value	β	(SE)	*p*-value	β	(SE)	*p*-value	β	(SE)	*p*-value	β	(SE)	*p*-value	β	(SE)	*p*-value
Demographics																		
Gender																		
Male (Ref)																		
Female	−0.55	(0.51)	0.28	−0.70	(1.53)	0.65	0.74	(0.39)	0.06	1.15	(0.89)	0.20	0.59	(0.73)	0.42	0.93	(1.71)	0.59
Race																		
White (Ref)																		
Other	−0.57	(0.47)	0.22	0.49	(1.34)	0.72	−0.32	(0.46)	0.49	−0.22	(1.04)	0.83	−0.05	(0.78)	0.95	−0.22	(1.63)	0.89
Marital Status																		
Married or living with partner (Ref)																		
Other	0.15	(0.53)	0.78	0.33	(1.53)	0.83	−0.04	(0.39)	0.91	−0.43	(0.87)	0.62	0.48	(0.77)	0.53	−0.02	(1.61)	0.99
Education																		
At least some college (Ref)																		
No college	−1.38	(0.56)	0.02	2.02	(1.61)	0.21	0.02	(0.37)	0.96	−0.20	(0.82)	0.81	0.57	(0.67)	0.39	2.20	(1.40)	0.12
Family Income																		
> = $75,000/year (Ref)																		
<$75,000/year	0.25	(0.48)	0.61	−0.59	(1.41)	0.67	−0.05	(0.37)	0.90	0.09	(0.83)	0.91	−0.46	(0.73)	0.53	0.06	(1.55)	0.97
Childcare Responsibilities																		
Some or Complete Responsibility (Ref)	−0.22	(0.47)	0.64	0.02	(1.36)	0.99	−0.07	(0.30)	0.81	0.89	(0.67)	0.19	0.35	(0.87)	0.69	0.83	(1.87)	0.66
None or Another Adult Responsible																		
Adult Care Responsibility																		
Responsible for at least one adult (Ref)	0.33	(0.47)	0.49	3.23	(1.35)	0.02	−0.10	(0.30)	0.74	0.21	(0.68)	0.76	−0.43	(0.54)	0.43	0.76	(1.16)	0.51
None																		
Health-Related Factors																		
Depressive Symptoms																		
<=1 day/week (Ref)																		
>1 day/week	0.25	(0.82)	0.76	−1.17	(2.34)	0.62	0.21	(0.45)	0.64	−0.65	(1.02)	0.53	−0.88	(0.99)	0.38	0.14	(2.05)	0.95
Hours Sleep																		
> = 6 h/night (Ref)																		
<6 h/night	0.32	(0.42)	0.45	0.08	(1.25)	0.95	−0.39	(0.33)	0.25	0.07	(0.77)	0.92	−0.97	(0.58)	0.10	−1.64	(1.28)	0.20
Leisure Time Physical Activity																		
None (Ref)																		
At least some	−0.14	(0.44)	0.75	0.44	(1.29)	0.73	0.35	(0.38)	0.35	−0.16	(0.90)	0.86	0.20	(0.60)	0.75	−0.30	(1.31)	0.82
Musculoskeletal Pain																		
None or Mild (Ref)																		
Moderate to Severe	−0.55	(0.42)	0.19	−0.09	(1.22)	0.94	0.00	(0.32)	1.00	0.09	(0.76)	0.91	−0.13	(0.63)	0.83	−0.67	(1.30)	0.61
Weight Perception																		
Interested in Changing (Ref)																		
Other	0.08	(0.40)	0.85	1.04	(1.21)	0.39	0.02	(0.32)	0.94	−0.19	(0.74)	0.79	−0.20	(0.57)	0.73	−0.03	(1.31)	0.98
Work-Related Factors																		
Civility Norms																		
High (>4) (Ref)																		
Low (<4)	0.42	(0.48)	0.38	1.14	(1.41)	0.42	0.20	(0.34)	0.57	−0.26	(0.78)	0.74	0.16	(0.71)	0.83	−0.83	(1.46)	0.57
Decision Latitude																		
Low (<3) (Ref)																		
High (>3)	0.09	(0.46)	0.85	−2.16	(1.36)	0.12	−0.52	(0.37)	0.16	−0.07	(0.86)	0.93	−0.53	(0.64)	0.41	0.39	(1.34)	0.77
Job Satisfaction																		
High (>4) (Ref)																		
Low (<4)	−0.29	(0.42)	0.49	−0.40	(1.23)	0.74	0.48	(0.34)	0.17	1.88	(0.79)	0.02	−0.17	(0.67)	0.80	0.51	(1.37)	0.71
Job Tenure																		
>5 years (Ref)																		
<5 years	0.11	(0.40)	0.78	0.78	(1.18)	0.51	0.54	(0.43)	0.21	1.03	(0.98)	0.29	0.70	(0.86)	0.42	1.60	(1.81)	0.38
Job Type																		
Administrative (Ref)																		
Floor	−0.08	(0.48)	0.86	1.56	(1.41)	0.27	0.35	(0.33)	0.30	1.19	(0.76)	0.12	0.30	(0.64)	0.64	−0.55	(1.43)	0.70
Procedural Justice																		
High (>4)																		
Low (<4)	−0.57	(0.45)	0.21	0.10	(1.33)	0.94	−0.31	(0.37)	0.40	0.47	(0.84)	0.57	0.15	(0.61)	0.81	0.01	(1.27)	1.00
Psychological Demands																		
Low (<3) (Ref)																		
High (>3)	0.45	(0.49)	0.36	−0.21	(1.44)	0.88	−0.55	(0.36)	0.12	−1.68	(0.83)	0.04	1.18	(0.75)	0.12	0.51	(1.66)	0.76
Social Support																		
Low (<3) (Ref)																		
High (>3)	−0.07	(0.46)	0.87	1.13	(1.38)	0.42	−0.21	(0.34)	0.54	−1.33	(0.79)	0.09	0.95	(0.58)	0.10	1.09	(1.29)	0.40
Stress in General																		
Low (<1.5) (Ref)																		
High (>1.5)	−0.10	(0.49)	0.84	0.69	(1.46)	0.64	−0.33	(0.34)	0.33	−1.04	(0.77)	0.18	1.01	(0.63)	0.11	0.35	(1.31)	0.79
Work-Life Balance																		
Very/Completely Successful (Ref)																		
Not/Somewhat Succesful	0.27	(0.46)	0.56	−0.47	(1.43)	0.74	−0.19	(0.34)	0.58	−0.49	(0.79)	0.53	0.68	(0.58)	0.24	−0.07	(1.21)	0.96
Work Overtime																		
<24 h/month (Ref)																		
>24 h/month	0.25	(0.48)	0.60	−1.31	(1.43)	0.36	0.60	(0.32)	0.07	1.53	(0.74)	0.04	0.02	(0.62)	0.97	1.51	(1.33)	0.26
Work Shift																		
First (Ref)																		
Other	0.05	(0.57)	0.93	−0.78	(1.69)	0.64	0.29	(0.41)	0.48	0.39	(0.94)	0.68	0.06	(0.78)	0.94	−0.99	(1.69)	0.56
Work Time Standing																		
>30 % of time (Ref)																		
<30 % of time	−0.48	(0.46)	0.30	2.04	(1.37)	0.14	0.26	(0.37)	0.48	1.20	(0.85)	0.16	0.51	(0.59)	0.39	−1.06	(1.28)	0.41

## Discussion

The objective of this study was to identify factors associated with obesity among manufacturing workers of different ages that might inform future workplace interventions. Our study is unique in that wemeasured a variety of demographic, health-related, and work-related factors as well astwo indicators of obesity,BMI and BFP, across workers in a specific industry (manufacturing) and in different age groups who may have different developmental stressors and needs. Our findings support the notion described in the Social Ecological Model that obesity is a multifactorial disease with many contributing factorsthat may differ across a worker’s lifespan [8, 15].

The trends in BMI and BFP of our participants by age are consistent with the previous literature. Similar to published studies by Orpana et al. and Mozaffarian et al., we observed larger BMI and BFP for older participants in our study [38, 39]. Also consistent with previous studies, we observed that older participants had smaller or negative changes in BMI compared to the younger participants in our study sample, who tended to have increases in BMI and/or BFP between time 1 and time 2 of the study [18, 40]. Participants in our sample were, on average, overweight, with BMIs in the 25–30 kg/m^2^ range. Therefore, it could be desirable to intervene on factors associated with increased BMI or BFP in this population.

One of the few factors that we observed to be consistently associated with higher baseline BMI and BFPregardless of age wasinterest in changing weight: participants in our population who reported that they were “interested in changing” their weight had consistently higher baseline BMIs and BFPs than those who were satisfied with their current weights. A previous study by Tamers et al. reported similar findings [41]. This factor may be a beneficial to consider as part of workplace interventions for all age groups. Information on whether workers are interested in changing their weights may help to identify those who would most benefit from an obesity intervention. Based on the theory of the Transtheoretical Stages of Change Model, which posits that behavior modification is more likely to occur when participants are ready to change [42], we might expect that these participants reporting an interest in changing their weight would be more likely to reduce their BMIs or BFPs throughout the measurement period. Unfortunately, similarly to another finding by Tamers et al. [41], we did not identify weight perception as a factor associated with change in BMI or BFP in our analyses, indicating that intentions to change weight are not sufficient to actually affect these indicators of obesity.

The majority of the factors that we identified as being associated with increased baseline BMI and BFP differed by age. We observed that education, childcare responsibilities, social support, and overtime work were only associated with differences in baseline BMI and/or BFP in the <45 year age group, while stress in general was only associated with differences in baseline BMI and/or BFP in the 45–55 year age group and job tenure in the >55 year age group. It is possible that individuals are more susceptible to the effects of certain exposures at different times in their lives. For example, in the <45 year age category people had higher childcare responsibilities and this factor was associated with higher BMI. This is in line with other research such as a study of working mothers by Dugan which found that self-care behaviors (including physical exercise, healthy eating, and weight management) were associated withhaving availabletime and energy, resources that are often consumed by acumulative workload consisting of paid work plus home/family work [43]. The study concluded that an effective intervention for this population would be one that takes place early in the day (e.g., a morning exercise class), ensuring that time and energy resources do not become depleted before people have an opportunity to use them for self-care. Such findings emphasize the importance of considering age and its related circumstances when planning interventions around obesity, as individuals in different age groups may benefit from interventions focused on different factors.

We observed associations between work-related factors and BMI or BFP among participants across all age categories. Work-related factors can affect obesity through many pathways from directly impacting energy expenditure via physical work demands to indirectly by influencing workers’ diets or leisure time physical activity levels as a result of work scheduling or workplace stress [44]. Our results support the idea of performing obesity interventions within the workplace.

Many of the work-related factors included in this study were selected because of their potential influence on workplace stress. Previous studies have demonstrated that a worker’s experience of stress at work can be affected by many factors such as civility [45], decision latitude [46], job satisfaction [47], procedural justice [48], and psychological demands [49]. Exposure to stress at work can result in neuroendocrine dysregulation [16], and may also lead to unhealthy behaviors [17], both of which may affect BMI or BFP.

Some factors that have been identified as being associated with obesity in previous studies were not associated with BMI or BFP in the current study. For example, while we did not observe any association between BMI or BFP and work time sitting/standing, increased sitting has been associated with increased BMI in several previous studies of office workers (e.g.[50]). This may be one example of a factor that is more relevant to obesity among office workers than manufacturing workers. Even compared to other studies among blue collar workers, we did not always observe the same results; for example, Duffy et al. [19] reported that low physical activity levels were significantly associated with obesity among operating engineers, while we found no association between physical activity and BMI or BFP in the current study. Workers across industries may have exposures or responses to different factors that may contribute to weight gain [20]. Therefore, it is important to identify industry-specific factors associated with obesity when considering workplace interventions.

We observed few factors associated with change in BMI or BFP. This finding is consistent with the results of previous studies reporting that a variety of factors were not significantly associated with change in obesity (e.g. [18, 50, 51]). This may also explain why a recent systematic review reported that there was little evidence to inform interventions aimed at preventing obesity [3]. On one hand, this indicates that the factors we considered were not associated with increases in BMI or BFP from time 1 to time 2. But, such research also implies that it is difficult to identify factors associated with decreases in BMI or BFP that could be used for interventions.

We considered both BMI and BFP as indicators of obesity in this study because they may be characterizing obesity in different ways. BMI and BFP are not always correlated [52]; BMI incorporates total weight including muscle and fat mass, while BFP only considers body fat. As a result, BFP is expected to represent the health risks associated with obesity more accurately;however, BMI is more commonly used in the literature because it is easier to measure [52]. In our study, we observed differences in the factors associated with BMI compared to BFP. It may be important to consider factors associated with BFP as well as of BMI for future interventions.

Several strengths of this study should be noted. First, our study provided information specific to the manufacturing industry on factors associated withBMI and BFP by age, considering factors from multiple dimensions. Such information is needed in order to develop targeted, effective obesity interventions. The longitudinal design where factors were measured at time 1 and assessed in terms of their association with changes in BMI and BFP from time1 to 2allows for temporality to be established for change in BMI and BPF, and although the cross-sectional analyses prevent causality from being established, they serve to identify groups that have higher BMI and BFP and may therefore benefit most from interventions. Second, the study’s comprehensive consideration of multiple demographic, health-related, and work-related factors simultaneously allows for more accurate evaluation of associations and reduces multiple testing [53].

The results of this study must be taken with consideration for the study’s limitations. First, we were unable to include any measure of several important factors associated with obesity including diet or energy intake or chronic health conditions such as cerebrovascular disease or sleep apnea in our analyses. Therefore, none of our results are adjusted for the effect of these factors, and it may be possible that the pathways by which some of the demographic, health-related, or work-related factors identified in our study affect obesity go through diet or health conditions. In addition, our measures of physical activity may not have fully characterized each participant’s actual physical activity level. It is also possible that other factors were not included in our analyses that could have been associated with obesity. Second, it is possible that we had limited power to detect differences in obesity by some of our factors such as depressive symptoms where there was limited variability in responses. Third, we only considered one time period of approximately 33 months for change in BMI and BFP. It is possible that the factors associated with change in obesity are dependent on the time period between assessments of BMI or BFP.

## Conclusions

In conclusion, we identified associations between individual, health-related, and work-related factors and obesity that differed by age in a group of manufacturing workers. Such results support the use of age-specific intervention strategies around obesity. More work must be done to identify factors or strategies associated with changes in obesity over time.

## Abbreviations

BMI: Body mass index
BFP: Body fat percentage
